# Recent progress on the activation of the cGAS–STING pathway and its regulation by biomolecular condensation

**DOI:** 10.1093/jmcb/mjac042

**Published:** 2022-07-08

**Authors:** Xiaoyu Yu, Zhen Zhao, Zhengfan Jiang

**Affiliations:** Key Laboratory of Cell Proliferation and Differentiation of the Ministry of Education, School of Life Sciences, Peking University, Beijing 100871, China; Peking-Tsinghua Center for Life Sciences, Peking University, Beijing 100871, China; Key Laboratory of Cell Proliferation and Differentiation of the Ministry of Education, School of Life Sciences, Peking University, Beijing 100871, China; Peking-Tsinghua Center for Life Sciences, Peking University, Beijing 100871, China; Key Laboratory of Cell Proliferation and Differentiation of the Ministry of Education, School of Life Sciences, Peking University, Beijing 100871, China; Peking-Tsinghua Center for Life Sciences, Peking University, Beijing 100871, China

**Keywords:** innate immunity, cGAS–STING, cGAMP, manganese (Mn^2+^), sulfated glycosaminoglycans (sGAGs), biomolecular condensate, STING phase-separator

## Abstract

The cyclic guanosine monophosphate (GMP)–adenosine monophosphate (AMP) synthetase (cGAS)–stimulator of interferon genes (STING) pathway, comprising the DNA sensor cGAS, the second messenger cyclic GMP–AMP (cGAMP), and the endoplasmic reticulum (ER) adaptor protein STING, detects cytoplasmic double-stranded DNA (dsDNA) to trigger type I-interferon responses for host defense against pathogens. Previous studies defined a model for the allosteric activation of cGAS by DNA-binding, but recent work reveals other layers of mechanisms to regulate cGAS activation such as the phase condensation and metal ions, especially the discovery of Mn^2+^ as a cGAS activator. Activation of the 2′3′-cGAMP sensor STING requires translocating from the ER to the Golgi apparatus. The sulfated glycosaminoglycans at the Golgi are found to be the second STING ligand promoting STING oligomerization and activation in addition to 2′3′-cGAMP, while surpassed levels of 2′3′-cGAMP induce ER-located STING to form a highly organized ER membranous condensate named STING phase-separator to restrain STING activation. Here, we summarize recent advances in the regulation of cGAS–STING activation and their implications in physiological or pathological conditions, particularly focusing on the emerging complexity of the regulation.

## Introduction

The innate immunity detects and defends against the invasion of a broad range of pathogens, including bacteria, viruses, parasites, and fungi. Recognition of pathogens is mediated by the germline-encoded pattern recognition receptors, which recognize cognate pathogen-associated molecular patterns or damage-associated molecular patterns (Murphy et al., 2014). The cyclic guanosine monophosphate (GMP)–adenosine monophosphate (AMP) synthetase (cGAS)–stimulator of interferon genes (STING) pathway recognizes double-stranded DNA (dsDNA) released by infected pathogens or self-DNA mislocalized in the cytoplasm (Motwani et al., 2019). cGAS binds to dsDNA and becomes activated to synthesize the second messenger 2′3′-cyclic GMP–AMP (2′3′-cGAMP). The endoplasmic reticulum (ER) protein STING, also named MITA or ERIS (Zhong et al., 2008; Sun et al., 2009), detects 2′3′-cGAMP and undergoes a translocation from the ER to the Golgi, where it becomes oligomerized to recruit and activate the downstream kinase TANK-binding kinase 1 (TBK1). The phosphorylated STING by TBK1 scaffolds the interactions between interferon regulatory factor 3 (IRF3) and TBK1, allowing for the phosphorylation and activation of IRF3 by TBK1 (Zhang et al., 2019). IRF3 then moves into the nucleus to initiate the expression of various cytokines including type I-interferons (IFNs), which eventually induce the production of hundreds of interferon-stimulated genes for host defense (Ishikawa and Barber, 2008).

cGAS is a nucleotidyltransferase that is allosterically activated by dsDNA-binding and catalyzes the formation of 2′3′-cGAMP from adenosine triphosphate and guanosine triphosphate (GTP) (Civril et al., 2013; Diner et al., 2013; Gao et al., 2013b; Kranzusch et al., 2013; Sun et al., 2013; Wu et al., 2013; Zhang et al., 2013). Besides cytosol dsDNA, cytoplasmic manganese (Mn^2+^) released from membrane-enclosed organelle binds to cGAS to enhance the sensitivity of cGAS to dsDNA by several orders of magnitude upon viral infection (Wang et al., 2018). Cytoplasmic Mn^2+^ also triggers cGAS to synthesize cGAMP independent of dsDNA by a novel catalytic mode, initiating type I-IFN response and cytokine production without any infection (Zhao et al., 2020). Therefore, Mn^2+^, as a powerful agonist of cGAS–STING, is now used in the antitumor treatments and vaccine adjuvants (Lv et al., 2020; Zhang et al., 2021).

STING (encoded by *STING1*), an ER-transmembrane protein, is the scaffold protein to recruit and activate downstream TBK1 and IRF3, leading to the expression of type I-IFNs and multiple pro-inflammatory cytokines (Ishikawa and Barber, 2008; Zhong et al., 2008; Sun et al., 2009). To be activated, STING undergoes a conformational change from ‘open’ to ‘close’, and then translocates to the Golgi apparatus to be oligomerized or polymerized (Shang et al., 2019; Zhang et al., 2019). Oligomerized STING recruits TBK1 to form STING–TBK1 oligomers to activate TBK1 by trans-phosphorylation, followed by TBK1 phosphorylation on STING and IRF3 to induce type I-IFNs and other cytokines (Shang et al., 2019; Zhang et al., 2019). cGAMP-bound STING exits ER via coat protein complex-II (COP-II) vesicles to translocate to the Golgi and trans-Golgi network (TGN). At the Golgi and TGN, STING oligomerizes with the help of sulfated glycosaminoglycans (sGAGs) and gets palmitoylated further (Fang et al., 2021; Taguchi et al., 2021). The coat protein complex-I (COP-I) vesicles regulate STING Golgi-to-ER retrograde traffic (Taguchi et al., 2021).

Biomolecular condensates are non-membrane-surrounded compartments in eukaryotic cells that allow molecules to be concentrated within condensates while still exchanging with the surroundings (Nott et al., 2015; Zeng et al., 2016; Banani et al., 2017; Boeynaems et al., 2018; Wang and Zhang, 2019). The dynamic of molecules and kinetics of biochemical reactions in the condensates can be regulated in a physical manner, which is different from classic membrane-surrounded organelles (Brangwynne et al., 2009; Li et al., 2012; Patel et al., 2015; Shin and Brangwynne, 2017). Recently discovered cGAS–DNA droplets and STING ER membranous biocondensates indicate that biomolecular condensation is a new mechanism modulating cGAS–STING signaling. Cytosol DNA-induced cGAS liquid-like droplets display enhanced cGAS activity (Du and Chen, 2018). On the contrary, continuously produced cGAMP by activated cGAS induces ER-resident STING to form an ER membranous condensate with a highly organized puzzle-like structure. Moreover, the STING condensate quickly undergoes a gel-like phase transition to isolate cGAMP-bound STING and TBK1 from IRF3, thus preventing STING from overreaction (Yu et al., 2021). The puzzle-like membranous structure of STING condensate highly resembles cubic membranes, a type of widely reported membrane structure that has been discovered for more than 60 years with only few clues about its initiation and functions (Yu et al., 2021).

In this review, we focus on the recent discoveries regarding the molecular mechanism of cGAS–STING activation and regulation, with a special focus on the cGAS activation by DNA and Mn^2+^, the STING ER–Golgi translocation and its activation by sGAGs, and the formation and function of STING membranous biomolecular condensates.

## cGAS activation by dsDNA

**Figure 1:**
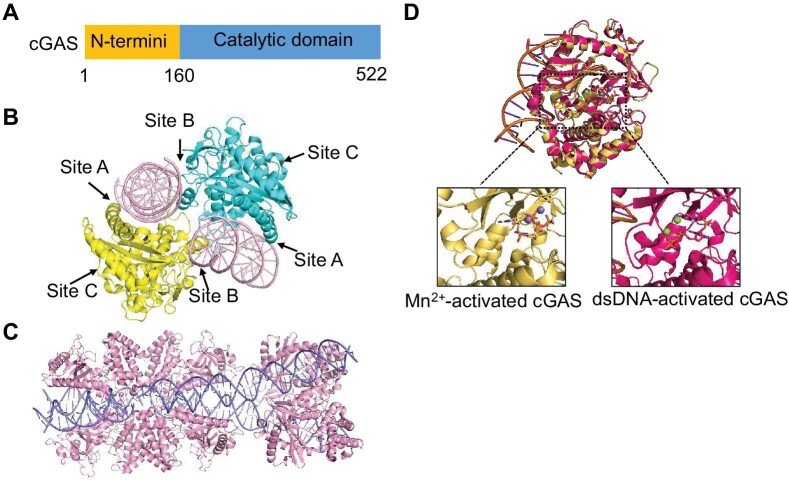
Activation of cGAS by DNA and Mn^2+^. (**A**) cGAS contains a disordered N-terminus and a catalytic domain. (**B**) The crystal structure of cGAS–DNA complex with three DNA-binding sites labelled (PDB: 4LEZ). (**C**) The crystal structure of oligomeric cGAS bound to a long dsDNA, forming a ladder-like structure (PDB: 5N6I). (**D**) The crystal structure of dsDNA-activated cGAS (red, PDB: 4K98) and Mn^2+^-activated cGAS (yellow, PDB: 7BUJ). Conformation changes of Mn^2+^-activated cGAS are overall similar to that of dsDNA-activated cGAS but form a unique η1 helix at the activation loop to empty the catalytic pocket, thus rendering it catalytically competent.

cGAS is constituted by a disordered amino-terminal stretch and a conserved Mab21 domain (residues aa 161–512 in humans) (Figure 1A). The Mab21 domain belongs to the nucleotidyltransferase superfamily (Kuchta et al., 2009), mediating dsDNA recognition and cGAMP synthesis (Civril et al., 2013; Gao et al., 2013c; Sun et al., 2013). The Mab21 domain consists of an N-lobe with a Rossman fold for substrate-binding and catalysis and a C-lobe bundle with a zinc-finger for dsDNA-binding and dimerization (Civril et al., 2013; Gao et al., 2013c; Sun et al., 2013). The catalytic domain of cGAS has three major DNA-binding sites (A, B, and C) (Figure 1B). DNA-binding site A recognizes dsDNA and induces the conformational switch to activate cGAS (Li et al., 2013; Zhang et al., 2014). The site B of cGAS binds to dsDNA and facilitates the formation of the 2:2 dimer complex (Li et al., 2013; Zhang et al., 2014). The 2:2 dimer complex is further stabilized by the interaction between zinc-fingers from each cGAS protomer, a process required for the enzymatic activation of cGAS (Civril et al., 2013). The site C promotes multivalent interaction among cGAS and DNA, thus promoting cGAS droplet formation and cGAMP production (Xie et al., 2019).

However, dsDNA less than ∼30 base-pairs is unable to activate human cGAS in cells (Civril et al., 2013; Gao et al., 2013b; Li et al., 2013; Zhang et al., 2014), indicating that cGAS activation by dsDNA is length-dependent. The cGAS dimers can prearrange the flanking DNA to promote subsequent binding of more cGAS dimers to form a ladder-like structure and further promote cGAS activation by DNA (Andreeva et al., 2017; Figure 1C). Mutations in cGAS are rapidly accumulated during evolution. There are <60% of amino-acid identity between human and mouse cGAS. Interestingly, mouse cGAS is quite sensitive to shorter DNA species compared to human cGAS (Zhou et al., 2018). Structural and biochemical comparisons between human and mouse cGAS unveil two residue substitutions specific in human cGAS, K^187^ and L^195^, which are critical in determining the preference of human cGAS for longer DNA species (Zhou et al., 2018). Mouse cGAS with human-like N^172^K and R^180^L mutations are sufficient to gain DNA-length specificity and a restrained cGAMP production like human cGAS, while human cGAS with mouse-like L^187^N and L^195^R mutations is able to be activated by shorter DNA and has a higher enzyme activity as mouse cGAS. The results reveal a species-specific regulation of cGAS activities by several nucleic acids (Zhou et al., 2018). This mode of cGAS activation probably provides a safeguard mechanism in human cells to avoid spurious cGAS activation, as cGAS is triggered only when the long-length dsDNA exceeds a concentration threshold.

## cGAS activation by Mn^2+^

Metal ions are critical components or regulators of nearly half of all enzymes and play pivotal roles in almost every aspect of biological processes. The important role of metal ions in immune responses and cancer therapies is receiving more and more attention and is one of the promising future directions in cancer immune. Therefore, metalloimmunology and metalloimmunotherapy have been termed recently to highlight the pivotal role of metal elements in immune responses as well as their cutting-edge applications in cancer immunotherapy (Li et al., 2019; Moon et al., 2021; Sun et al., 2021a). Particularly, divalent cations including Ca^2+^, Zn^2+^, and Mn^2+^ have been demonstrated to be involved in the regulation of the cGAS–STING pathway. Ca^2+^ is reported to regulate STING through the Ca^2+^ sensor stromal interaction molecule 1 (STIM1), which restrains STING to the ER (Srikanth et al., 2019). The STIM1 deficiency disrupts Ca^2+^ homeostasis and promotes the ER-to-Golgi translocation of STING, causing an enhanced STING activation (Srikanth et al., 2019). Recently, Du and Chen (2018) reported that Zn^2+^ increases cGAS activation by promoting the phase separation of cGAS–DNA complex. TPEN, a Zn^2+^ chelator, inhibits cGAS activation in cells, but it is noteworthy that TPEN is also able to chelate Fe^2+^, Mn^2+^, and some other divalent cations (Shumaker et al., 1998). Wang et al. (2018) found that viral infection induces the release of Mn^2+^ ions from membrane-enclosed organelles such as mitochondria and the Golgi into the cytosol. Accumulation of Mn^2+^ in cytosol tremendously enhances the sensitivity of cGAS to dsDNA and augments the binding affinity of STING to cGAMP, thus promoting the activation of the cGAS–STING pathway. Moreover, treatment of cells or mice with Mn^2+^ (such as MnCl_2_) can stimulate them into an antiviral state through the cGAS–STING-dependent type I-IFN responses. The dietary-induced Mn-insufficient mice produce less cytokines and are more vulnerable to DNA viruses as STING-deficient (*Sting1 ^−/^^–^*) mice, reinforcing the critical role of Mn^2+^ in cGAS–STING activation upon DNA virus infection (Wang et al., 2018).

Importantly, physiological amount of Mn^2+^ activates cGAS mutants, which are defective in dsDNA/dimerization-dependent activation, both *in vitro* and in cells, indicating that Mn^2+^ activates monomeric cGAS (Hooy et al., 2020). Structural analysis reveals that the Mn^2+^-activated cGAS undergoes overall similar conformational changes to dsDNA-activated cGAS, but forms a unique η1 helix at the activation loop to empty the catalytic pocket, thus rendering it catalytically competent (Figure 1D). Moreover, in Mn^2+^-activated cGAS structures, the two linear intermediates pppG[2′-5′]pG and pG[2′-5′]pA bind to the active site in an inverted orientation compared to those in DNA-activated cGAS structures, suggesting a non-canonical cGAMP synthesis without substrate flip-over, which may explain the significantly accelerated activity of cGAS in the presence of Mn^2+^ (Wang et al., 2018; Hooy et al., 2020). Unlike the two Mg^2+^ in DNA-activated cGAS, two catalytic Mn^2+^ are found to bind to the triphosphate moiety of the inverted substrate, but without coordination by the acidic catalytic triad residues (Zhao et al., 2020). Therefore, changes in cytoplasmic concentration of Mn^2+^ may be a second messenger used by innate immune cells for their intracellular communication. Additionally, cGAS is found to preferentially utilize Mn^2+^ over Mg^2+^ for its catalytic activity, and Mg^2+^ facilitates Mn^2+^ utilization rather than competes against it (Hooy et al., 2020).

Mn^2+^-mediated STING activation is also important for the host defense against bacterial infections. A bacterial type VI secretion system (T6SS)-secreted 48-residue micropeptide crucial for the pathogenesis of *Yersinia pseudotuberculosis* is identified to inhibit c-di-GMP-triggered STING activation. The micropeptide inhibits STING signaling by chelating Mn^2+^ in host cells, mediating a previously unrecognized immune evasion strategy by bacteria (Zhu et al., 2021). Importantly, similar T6SS clusters containing putative Mn^2+^-binding effectors have been identified in diverse bacterial strains, suggesting that the Mn^2+^ sequestration mechanism might be employed by a broad range of pathogenic bacteria to suppress host innate immune responses.

## Applications of Mn^2+^-activated cGAS–STING signaling

The ability of Mn^2+^ to activate cGAS-dependent signaling makes it applicable to develop Mn^2+^-based antimicrobial/antitumor drugs or immune adjuvants for vaccines (Van Herck et al., 2021). The nanoparticle and liposome-based Mn^2+^ delivery strategies are of particular interest (Hou et al., 2020; Wang et al., 2021c). In fact, as a natural micronutrient necessary for diverse biological activities, Mn^2+^ facilitates antigen uptake and presentation by the antigen-presenting cells (APCs), as well as germinal center formation in the adaptive immune responses (Zhang et al., 2021). A colloidal manganese salt (Mn jelly, MnJ) is formulated and proves to be an effective adjuvant for stimulating both humoral and cellular immune responses (Zhang et al., 2021). MnJ also functions as a mucosal adjuvant to promote secreted IgA production via intranasal immunization. The adjuvant effects of MnJ have been proved for various antigens, including proteins/peptides as T cell-dependent antigens and T cell-independent antigens such as the bacterial capsule polysaccharides, indicating that MnJ is a potent universal immune adjuvant (Zhang et al., 2021). Importantly, besides MnJ, various Mn^2+^-containing nanoparticles have been formulated and significantly improved the efficacy of vaccines against influenza virus (Cui et al., 2021), hepatitis B virus (Lin et al., 2021), rabies virus (Wang et al., 2021c), and novel coronaviruses (Sun et al., 2021b; He et al., 2021; Wang et al., 2021b) in different animal models.

Immune checkpoint molecules, such as the well-known PD-1 and PD-L1, are essential as brakes to prevent the overactivation of T cell-mediated immune responses. However, tumor cells harness these inhibitory mechanisms to escape the immune surveillance (Ishida et al., 1992; Freeman et al., 2000; Ahmadzadeh et al., 2009). The immune checkpoint inhibitors boost the immune response. Unfortunately, only ∼20% of tumor patients respond to the immunotherapies with high efficacies, presumably due to inefficient activation of the innate immune responses (Topalian et al., 2015; Ribas et al., 2016; Huang et al., 2017). The cGAS–STING pathway has gained enormous interests in the field of immuno-oncology. Activation of this pathway can drive both innate and adaptive immune responses by promoting APC cell activation and the cross-priming of T cells (Yum et al., 2019), especially among immunosuppressive tumor microenvironments. Lv et al. (2020) reported that Mn^2+^ is critical for the innate immune system to surveil the tumor cells, as Mn-insufficient mice exhibit less-controlled tumor growth and metastasis than normal mice, with a decreased level of tumor-infiltrating CD8^+^ T and NK cells. More importantly, when administrated intranasally, intravenously, or intratumorally, Mn^2+^ induces robust and systemic antitumor responses in several mouse models (lung metastatic melanoma B16F10, colon adenocarcinoma MC38, Lewis lung carcinoma LLC, and T lymphoma E.G7) (Lv et al., 2020). The antitumor responses are also exhibited in multidrug (immuno)-resistant cancer patients in a phase I clinical trial (Lv et al., 2020). In these mouse models, Mn^2+^ treatments effectively promote NK cell function, DC/macrophage maturation/activation, and CD8^+^ T cell differentiation/activation to suppress the tumor growth and metastasis (Lv et al., 2020). Mn^2+^ also exhibits a more efficient antitumor effect when combined with anti-PD-1 antibody therapy (Lv et al., 2020). Moreover, in patients with advanced metastatic solid tumors who failed in standard anticancer treatments such as chemotherapies, radiotherapy, and immunotherapies, Mn^2+^ administration exhibits a notably improved therapeutic efficacy with 45.5% objective response and 90.9% disease control (Lv et al., 2020). Significantly, similar enhanced antitumor effects by Mn^2+^-promoted cGAS–STING activation in various antitumor immunotherapies have been reported (Hou et al., 2020; Chen et al., 2021; Gao et al., 2021; Li et al., 2021a; Liu et al., 2021; Song et al., 2021; Sun et al., 2021a; Wang et al., 2021a; Yang et al., 2021; Yi et al., 2021). Considering the component simplicity and steadiness of Mn^2+^, Mn^2+^ treatment or combined antitumor therapies including immunotherapies may have promising clinical potential.

However, the actions of Mn^2+^ in physiological and pathological contexts, especially in immunity, remain to be fully understood. Unlike Mg^2+^, which is abundant in cytoplasm, cellular Mn^2+^ is mainly restrained in membrane-coated compartments like the Golgi apparatus and mitochondria (Martinez-Finley et al., 2013; Carmona et al., 2014). Viral or bacterial infection liberates Mn^2+^ from mitochondria and the Golgi to be accumulated in the cytoplasm, but the underlying mechanisms of Mn^2+^ release remain to be fully elucidated. Furthermore, there is no chemical tools available for the non-invasive and quantitative detection of Mn^2+^ in living cells or tissues now. Therefore, developing new tools and techniques to trace the location/distribution and dynamic changes of the labile Mn^2+^ pool in different cellular compartments is imperative. Moreover, the potential toxic effect of Mn^2+^ in human use including neurotoxicity should also be carefully evaluated.

## Regulation of cGAS activation by biomolecular condensation

**Figure 2:**
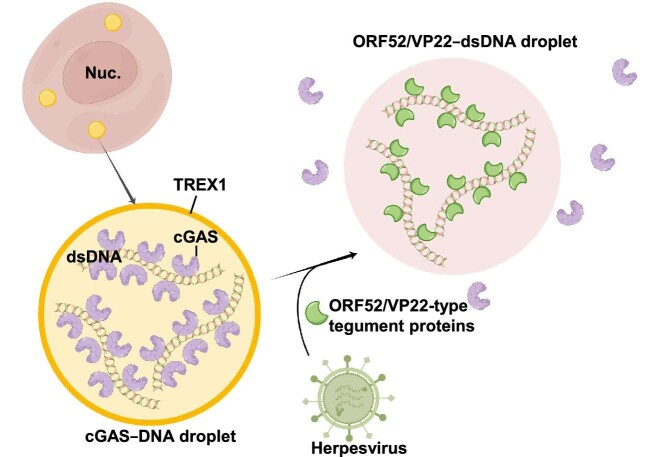
cGAS activation is regulated by biomolecular condensation. Cytosolic dsDNA substantially induces liquid–liquid phase separation of cGAS–DNA to form liquid-like droplets that promote cGAS activation and 2′3′-cGAMP production. TREX1, a cytosolic exonuclease that digests DNA, is selectively restrained to the external surface of the cGAS droplet, thus suppressing the DNA degradation within droplets and prolonging the activation of cGAS by DNA. Formation of cGAS–DNA droplets can be interfered by viral tegument factors, such as ORF52 and VP22 proteins from herpes viruses. Viral tegument factors bind to dsDNA and replace cGAS in the droplet, thus effectively inhibiting cGAS activation and subverting host defense.

Moreover, the cytosolic dsDNA, especially long DNA molecules, substantially induce liquid–liquid phase separation of cGAS–DNA to form liquid-like droplets that promote cGAS activation and 2′3′-cGAMP production (Du and Chen, 2018). The disordered and positively charged N-terminus of cGAS binds to dsDNA and is critical for the cGAS–DNA phase separation (Wang et al., 2017; Du and Chen, 2018). Three-prime repair exonuclease 1 (TREX1) is a cytosolic exonuclease that digests DNA. Its deficiency causes the accumulation of self-DNA in the cytoplasm, which leads to severe autoimmune diseases mainly through aberrant cGAS–STING activation (Yan et al., 2010; Yan, 2017). The cGAS droplet is also shown to selectively restrain TREX1 to the external surface of the droplet, thus suppressing the DNA degradation by TREX1 and prolonging the activation of cGAS by DNA (Zhou et al., 2021). Interestingly, viral tegument factors, such as ORF52, VP22, and ORF9 proteins from herpes viruses, are demonstrated to interfere with the cGAS–DNA droplet formation by binding to dsDNA and replacing cGAS in the droplet, which effectively inhibit cGAS activation and subvert host defense (Xu et al., 2021; Figure 2).

## STING activation by cyclic dinucleotides

The 2′3′-cGAMP produced by cGAS, as well as other cyclic dinucleotides (CDNs) from invaded bacteria, is detected by the ER-localized transmembrane protein STING. The C-terminal domain of STING containing the ligand-binding domain (LBD) and the cytosol-facing C-terminal tail (CTT) recognizes and binds to cGAMP. Cryo-electron structures (cryo-EM) of full-length STING proteins from human or chicken in the apo or 2′3′-cGAMP-bound states are recently resolved. The structures reveal a topology of the N-terminal transmembrane segment of STING, in which STING spans the ER membrane for four times. In the apo state, inactive STING molecules reside as dimers through interactions at the transmembrane regions as well as the LBD, with its LBD domains forming a V-shaped structure for ligand binding (Huang et al., 2012; Ouyang et al., 2012; Shang et al., 2012; Yin et al., 2012; Zhang et al., 2013). Upon binding to cGAMP, the lid of the LBD closes, leading to a 180° rotation of the LBD around the transmembrane domain. The rotation is coupled with conformational changes of the LBD to promote STING oligomerization in a side-by-side manner. The oligomerization of STING is required for its activation by CDNs.

Oligomerized STING recruits TBK1 to initiate downstream signaling. The CTT of STING contains a highly conserved TBK1-binding motif as well as a pLxIS motif, which is necessary for IRF3 recruitment and activation (Liu et al., 2015; Zhao et al., 2016). Recent cryo-EM structure of full-length chicken STING in complex with human TBK1 further unveils that the STING CTT has a β-strand-like conformation. The CTT is inserted into the groove between the kinase domain of one TBK1 and the scaffold and dimerization domain of another neighboring TBK1 (Zhang et al., 2019).

Interestingly, the phosphorylation site S^366^ of STING by TBK1 is far away from the active site of the directly associated TBK1, but can engage with TBK1 that binds to the adjacent STING (Zhang et al., 2019). In addition, there is minimal conformational change between the kinase domains of apo-TBK1 and TBK1 in complex with STING, suggesting that TBK1 is not activated by conformational changes in the kinase domain upon binding to STING (Tu et al., 2013; Zhang et al., 2019). Due to geometric constraints, the kinase domain of TBK1 normally cannot phosphorylate S^172^ in its activation loop to get activated, regardless of two TBK1 molecules are bound in close distance on one STING dimer (Larabi et al., 2013; Tu et al., 2013; Zhang et al., 2019). Besides, co-immunoprecipitation and imaging studies suggest that the inactive STING dimers on the ER have already recruited some TBK1 without TBK1 phosphorylation (Zhang et al., 2019). All these results suggest that STING phosphorylation by TBK1 requires the oligomerization of both STING and TBK1 molecules. The binding of TBK1 to STING oligomers induces the clustering of TBK1 and trans-autophosphorylation of TBK1. The TBK1 that is bound to the two CTT of one STING dimer can phosphorylate S^366^ of neighboring STING proteins that are not bound to TBK1 (Zhang et al., 2019). Activated STING and TBK1 then recruit and phosphorylate IRF3.

## The Golgi translocation-dependent STING activation

The translocation of STING from the ER to the Golgi is required for STING activation by CDNs. Blocking STING translocation by *Shigella* effector IpaJ or Brefeldin A entirely inhibits the downstream signaling and cytokine production, regardless of the cGAMP is continuously produced by activated cGAS (Ishikawa et al., 2009; Konno et al., 2013; Dobbs et al., 2015). The COP-I and COP-II vesicles mediate the cargo trafficking between the ER and the Golgi and are thought to mediate the trafficking of STING as well. Particularly, COP-II is needed for vesicles sprouting from the ER to translocate to the Golgi (Barlowe and Helenius, 2016). The COP-II complex is composed of Sar1 (a small GTPase), Sec23, Sec24, Sec13, and Sec31. Knockdown of Sar1A and Sar1B (mammalian paralogs of Sar1), Sec13, Sec23, Sec24, or Sec31 impairs the translocation of STING from the ER to the Golgi and activation of STING, suggesting a critical role of COP-II vesicle-mediated translocation in STING activation (Ogawa et al., 2018; Sun et al., 2018; Gui et al., 2019; Ran et al., 2019). Additionally, ZDHHC1 (Zhou et al., 2014) and iRhom2 (Luo et al., 2016) have been reported to positively regulate STING translocation and activation. Eventually, the activated STING molecules are translocated to the lysosome for degradation (Gonugunta et al., 2017).

Compared to the COP-II complex that mediates the protein translocation from the ER to the Golgi, COP-I complex mediates the retrograde Golgi-to-ER translocation (Letourneur et al., 1994; Scales et al., 1997). Recently, it has been reported that the mutation of COPA gene, which encodes COP-α of the COP-I complex, causes constitutive STING ER-exit and activation (Deng et al., 2020; Lepelley et al., 2020; Mukai et al., 2021; Steiner et al., 2022). Heterozygous missense mutation in WD40 domain of the COPA gene is related to the COPA syndrome, which is a Mendelian syndrome with autoimmune disorder featured with interstitial lung disease, joint inflammation, and elevated type I-IFN signaling (Watkin et al., 2015; Volpi et al., 2018). With the expression of autoimmune disease-related COP-α variants, STING cannot be retrieved back to the ER but accumulates on the Golgi. High concentration of STING molecules on the Golgi directly induces cGAS-independent but palmitoylation-dependent STING activation at the TGN (Mukai et al., 2021). SURF4, a protein that circulates between the ER/ER–Golgi intermediate compartment (ER/ERGIC), is an adaptor protein that facilitates COPA-mediated retrograde translocation (Deng et al., 2020; Mukai et al., 2021). Upon viral infection, cGAMP efficiently impairs the formation of STING/Sur4/COP-α complex, releasing STING from ER retrogradation. All these results support a notion that STING can be activated without cGAMP binding and factors in the Golgi drive STING activation, which is consistent with the role of sGAGs as the second STING ligand (see below).

Niemann–Pick type C1 (NPC1), a lysosomal membrane protein, is recently identified to recruit STING to the lysosome after STING activation at the Golgi. NPC1 deficiency enhances STING signaling by promoting STING accumulation at the Golgi via SREBP2–SCAP and impairing degradation of activated STING (Chu et al., 2021). In the steady state, TOLLIP is found to stabilize STING from degradation by direct interaction (Pokatayev et al., 2020).

## STING activation by sGAGs in the Golgi

However, it remains unclear why STING activation requires its translocation from the ER to the Golgi. Recently, sGAGs synthesized in the Golgi apparatus are demonstrated to be critical for STING oligomerization and activation. Through a genome-wide CRISPR–Cas9 screen, genes involved in the GAG biosynthesis and sulfation are identified to be critical for STING activation (Fang et al., 2021).

Previous work demonstrated that negatively charged sulfate groups in sGAGs provide a great multivalent landing plug for proteins through electrostatic interactions. sGAGs are also known to interact with various proteins via binding to and neutralizing positively charged or polar residues of targeted proteins and to facilitate them to polymerize (Soares da Costa et al., 2017; Smock and Meijers, 2018). Similarly, STING binds to sulfated GAGs through its luminal, positively charged and polar residues. These residues are evolutionarily conserved, and selective mutation of specific residues inhibits STING activation (Fang et al., 2021). Furthermore, purified or chemically synthesized sGAGs can directly induce the oligomerization and activation of STING *in vitro* (Fang et al., 2021). Thus, sGAGs in the Golgi are necessary and sufficient to drive STING oligomerization and activation.

The chain-length and *O*-linked sulfation of sGAGs impact the level of STING oligomerization and, thereby, its activation (Fang et al., 2021). Deficiency of SLC35B1, which transports the sulfur donor into the Golgi for GAG sulfation, impairs STING activation and host defense to DNA viral infection. Interestingly, sGAGs are also important for the accumulation of STING at the Golgi after cGAMP stimulation (Fang et al., 2021). It was reported that STING becomes oligomerized and autoactivated when accumulated in the Golgi apparatus (Mukai et al., 2016; Deng et al., 2020; Lepelley et al., 2020). sGAGs, which are synthesized in the Golgi (Vigetti et al., 2014), may activate STING via promoting STING retention on the Golgi as well as STING oligomerization. The sGAGs-driven STING activation explains how STING oligomerization is regulated and provides a mechanistic understanding of the Golgi translocation-dependent STING activation.

In addition, previous reports demonstrate that STING palmitoylation at C^88^ and C^91^ also promotes STING oligomerization by allowing STING to cluster into lipid rafts at the TGN (Linder and Deschenes, 2007; Mukai et al., 2016; Taguchi et al., 2021). Mutation of C^88^ and C^91^ or treating cells with the palmitoylation inhibitor 2-bromopalmitate inhibits STING palmitoylation and IRF3 phosphorylation (Mukai et al., 2016). However, STING translocation from the ER to the Golgi is not affected in the STING C^88^/C^91^-mutant cells (Mukai et al., 2016). It is conceivable that STING palmitoylation facilitates its clustering into lipid rafts on the Golgi from STING cytosolic side whereas sGAGs induce STING oligomerization from STING luminal side, altogether leading to the full activation of STING and TBK1.

Notably, STING–sGAGs binding is probably regulated by levels of acidification. It has been well documented that after CDN binding, STING translocates from the ER to the perinuclear compartments that include the Golgi, endosomes, and autophagy-related compartments, while intracellular sGAGs are compartmentalized into organelles like the Golgi, secretory vesicles, and endosomes (Kolset et al., 2004). Interestingly, acidification develops along with the ER (pH = 7.0), the Golgi complex, and reaches its peak in the TGN (pH = 6.0) (Kim et al., 1998; Llopis et al., 1998). Since the imidazole group of histidine (H^42^ in human STING and H^50^ in mouse STING) is positively charged at lower pH, the high affinity of sGAGs and STING is only found at the acidic Golgi (Fang et al., 2021). The pH-regulated STING–sGAGs interaction may explain why STING oligomerization happens mainly on the TGN and demonstrate an elaborate regulation of STING activation along with the Golgi apparatus translocation.

Recently, cryo-EM structure of STING and a small-molecule compound C53 reveals a novel binding pocket of STING in its transmembrane domain. C53 promotes STING oligomerization by binding to an undiscovered pocket formed among two TM2 and two TM4 helices of two STING protomers (Lu et al., 2022). The residues H^50^ S^53^ in TM2 and Y^106^ M^120^ in TM4 are critical to cGAMP–C53-induced STING oligomerization (Lu et al., 2022). Notably, these luminal residues in the transmembrane domain binding pocket are also critical for sGAGs–STING interaction, and sGAGs have been proved to promote STING oligomerization upon viral infection, as the second STING ligand (Fang et al., 2021). Thus, it is quite possible that sGAGs are the natural ligand of the newly discovered STING binding pocket.

**Figure 3:**
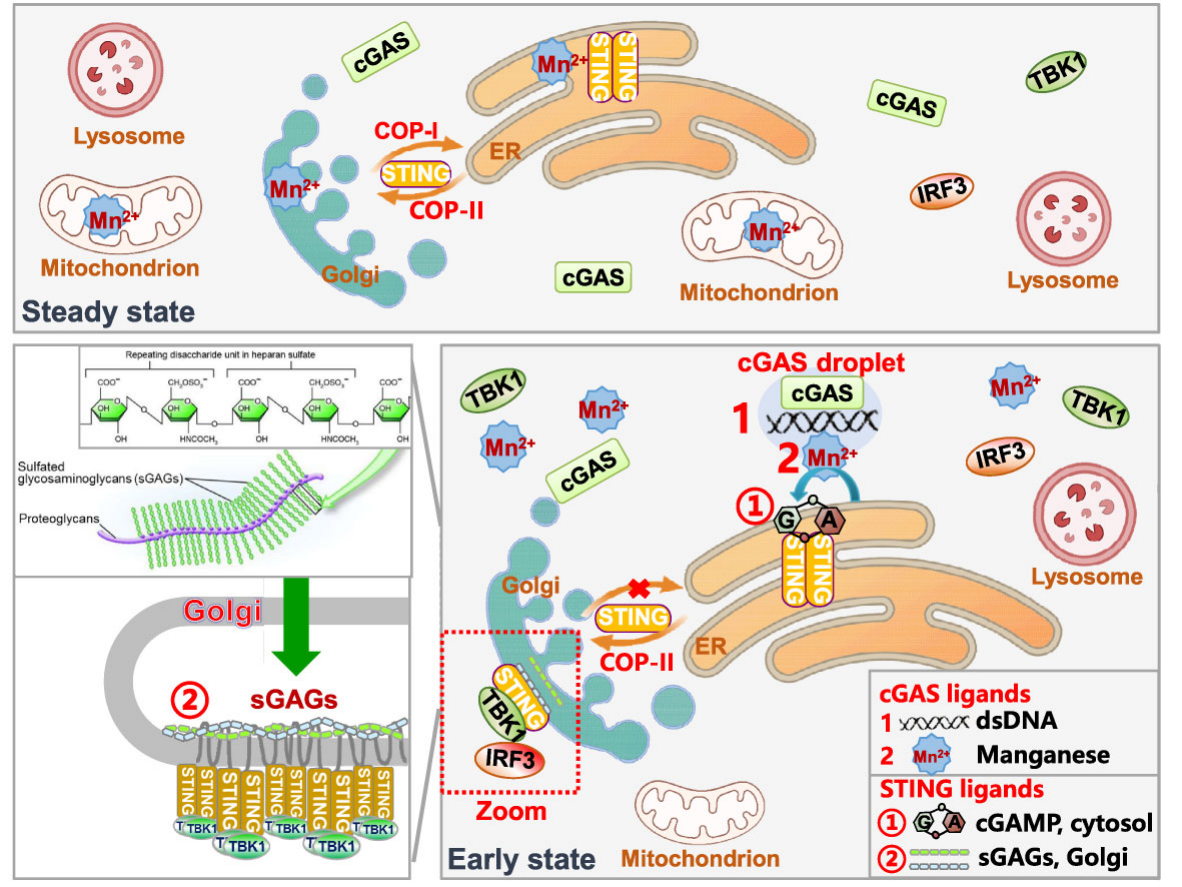
The cGAS–STING pathway at steady state and early state of activation. A schematic illustrates the process of dsDNA-induced cGAS–STING activation. At a steady state, STING constantly translocates to the Golgi via COP-II vesicles but is immediately retrieved back to the ER by COP-I vesicles. Upon viral infection, cGAS activated by the cGAS ligand, the cytoplasmic dsDNA, starts to synthesize the STING ligand 2′3′-cGAMP. Multivalent interactions among cGAS and long DNA molecules induce liquid-like cGAS–DNA droplet formation to facilitate cGAS activation. Mn^2+^, as a second cGAS ligand, is released from membrane-enclosed organelles and accumulates in the cytoplasm upon infection. Mn^2+^ promotes cGAS activation by enhancing the sensitivity of cGAS to dsDNA. cGAMP interrupts the STING Golgi-to-ER retrogradation, so STING dimer is released to the Golgi. At the Golgi apparatus, the lower pH value enhances interaction between the second STING ligand sGAGs and STING by electrostatic interaction, promoting STING accumulation and oligomerization. Oligomerized STING recruits TBK1 and promotes TBK1 oligomerization and autophosphorylation, which in turn phosphorylates STING at S^366^. Activated STING–TBK1 oligomers then recruit and phosphorylate IRF3 to initiate the expression of various cytokines.

Collectively, it is likely that STING dimers are continuously transported to the Golgi and TGN from the ER by the COP-II complex. However, in a steady state, when the formation of STING/SURF4/COP-α axis is not impaired by cGAMP, STING proteins transported to the Golgi are immediately caught back by the COP-I complex, preventing STING from reaching the TGN and getting oligomerized. Upon cGAMP binding, STING released from Golgi-to-ER retrograde trafficking is able to translocate to the TGN, where the lower pH value enhances sGAGs–STING interaction and traps STING upon arrival. The dynamic equilibrium of STING ER–Golgi transportation regulates STING activation precisely (Figure 3).

## Negative regulation of STING by the STING phase-separator

Biomolecular condensation mediated by polyvalent interactions among molecules is another underlying mechanism modulating the cGAS–STING pathway. Biomolecular condensates are non-classic cellular compartments that concentrate molecules without a physical barrier, efficiently separating the interior components from surrounding material (Brangwynne et al., 2011; Banani et al., 2017; Boeynaems et al., 2018; Wang and Zhang, 2019). Usually, formation of biomolecular condensates is driven by weak multivalent intramolecular interactions provided by soluble proteins with modular domains or intrinsically disordered regions (IDR) (Li et al., 2012; Nott et al., 2015; Zeng et al., 2016). Biomolecular condensates often become viscoelastic, transforming from a liquid-like state into a gel-like state that stops exchanging molecules with the outside (Feng et al., 2014; Lin et al., 2015; Harmon et al., 2017). Biomolecular condensates affect biological reaction kinetics and specificity via regulating protein concentration within condensate or molecular motion via viscoelasticity (Brangwynne et al., 2009; Shin and Brangwynne, 2017).

**Figure 4:**
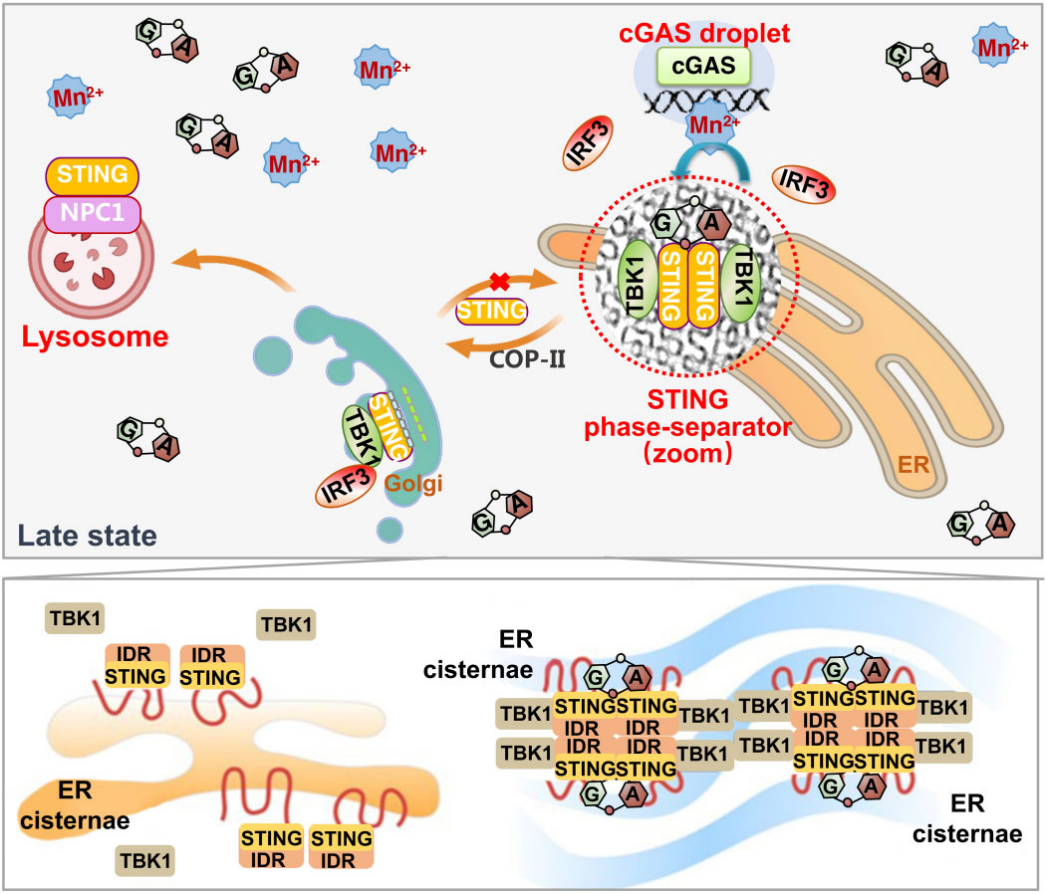
The cGAS–STING pathway at the late state of activation. A schematic illustrates the late state of cGAS–STING activation. At the end of STING activation, NPC1, a lysosome protein, recruits STING to the lysosome, where STING is degraded. Moreover, when continuously accumulated cytosol 2′3′-cGAMP exceeds a concentration threshold, it initiates ER-resident STING to form the STING phase-separator, a membranous biomolecular condensate with a highly organized puzzle-like membranous structure. The STING phase-separator is organized by multivalent interactions among STING IDR regions (aa 309–342, bottom). It functions to suppress the cGAS–STING pathway by trapping cGAMP-bound STING and TBK1 in gel-like condensates, thus buffering the concentration of free cytosolic cGAMP, STING, and TBK1.

Apart from DNA-induced cGAS droplets (discussed above), cGAMP-induced ER membranous STING biomolecular condensate, which is named the STING phase-separator, is also reported recently (Yu et al., 2021). Upon infection of DNA virus, cGAMP produced by the activated cGAS first activates STING by promoting STING translocation from the ER to the Golgi. In the late stage of infection, however, continuously activated cGAS results in a constantly increased cGAMP concentration. When cGAMP levels exceed the threshold, ER-resident (untranslocated and unactivated) STING is induced to form a highly organized puzzle-like ER membranous biocondensate. The formation is supposed to be driven by both cGAMP-induced conformational change of STING dimers and weak intramolecular multivalent interactions among STING IDRs. It is hypothesized that such weak intramolecular interactions among STING proteins initially induce the formation of a highly fluidic membranous structure. The intramolecular interactions anneal or zipper ER cisternae inversely and transform the otherwise cytosolic side of cisternae into a restricted composition. With time, even higher levels of cGAMP further induce this membranous condensate to transit into a gel-like state, thus decreasing molecular dynamic within the condensate. In this way, the puzzle-like ER membranous STING phase-separator separates cGAMP-bound STING and TBK1 from their downstream protein IRF3, which prevents innate immunity from overactivation, presumably acting like a ‘STING–TBK1–cGAMP sponge’ (Figure 4).

2′3′-cGAMP activates STING potently. The affinity of 2′3′-cGAMP for human STING is very high, with a dissociation constant of 4.59 nM, which is further augmented by Mn^2+^. Theoretically, it should be degraded rapidly by their specific phosphodiesterases like other second messengers, such as cAMP/cGMP, c-di-GMP, c-di-AMP, and 3′3′-cGAMP, to ensure a well-controlled signaling. However, there is only little knowledge about how 2′3′-cGAMP concentrations are regulated within mammalian cells. In bacteria, there are three 3′3′-cGAMP-specific phosphodiesterases (V-cGAP1/2/3) in *Vibrio cholera* (Gao et al., 2015) and another one named PmxA in *Myxococcus xanthus* (Yadav et al., 2019). In mammals, only the extracellular matrix- and/or ER lumen-localized cGAMP hydrolase ENPP1 has been reported (Li et al., 2014), which indicates that 2′3′-cGAMP must be transported across the plasma membrane or the ER membrane for degradation. So far, no intracellular hydrolase degrading 2′3′-cGAMP has been discovered. It is likely that the ER membranous ‘STING–TBK1–cGAMP sponge’ may facilitate cGAMP ER-membrane transportation for ENPP1 degradation. Thus, this 2′3′-cGAMP-driven STING phase-separator may circumvent the requirement for the intracellular 2′3′-cGAMP hydrolase(s) to degrade the excessive 2′3′-cGAMP.

As the first reported highly organized membranous biocondensate formed by ER transmembrane protein, STING phase-separator is actually a discrete phase consisting of protein molecules and highly organized membranes. Multivalent interaction among STING molecules is only necessary but not sufficient for the formation of puzzle-like membranes. It is speculated that the highly condensed STING molecules may induce spontaneous membrane curvature (Stachowiak et al., 2012), together with weak antiparallel interaction among cytosolic domain (STING IDR), to induce the puzzle-like structure formation. Therefore, STING phase-separator is more like a ‘liquid crystal’ rather than a ‘liquid droplet’ referring to its physical properties.

Notably, the puzzle-like membrane structure in STING phase-separator looks like a type of membrane structure called cubic membranes. Cubic membranes (also termed honeycombed lamellae/cristae, puzzles tridimensionnels, organized smooth ER, tubuloreticular structures, cylindrical confronting cisternae, etc.) are highly curved, three-dimensional nanoperiodic membrane structures corresponding to mathematically well-defined triply periodic minimal surfaces (Chin et al., 1982; Yamamoto et al., 1996). Cubic membranes are possibly first described back in 1959 by Pappas and Brandt (1959) in the mitochondria of *Pelomyxa carolinensis* Wilson (*Chaos chaos* L.) and by Almsherqi et al. (2009) in the mitochondria of spermatids of *Euscorpius Flavicaudis*, followed by hundreds of papers demonstrating that they can actually emerge from almost any cytomembranes, including the ER, the nuclear envelope, mitochondrial membranes, and the Golgi complex. Actually, they have been observed in enormous types of cells across all kingdoms of life under different physiological or pathological conditions, especially in stressed, diseased, or virus-infected cells. However, knowledge about the formation and function of such structures in cells is very limited, and research so far remains descriptive (Almsherqi et al., 2009; Chong and Deng, 2012). Accordingly, these morphologies obtain >130 different nicknames (Almsherqi et al., 2009).

Importantly, cubic membranes have been frequently reported to occur in the pathogenesis of viral infection, neoplasia, muscular dystrophy, and many types of autoimmune diseases (Almsherqi et al., 2009). They are even regarded as an indicator of infection by HBV (Schaffner et al., 1977) and SIV/HIV (Grimley and Schaff, 1976; Kostianovsky et al., 1987; Lee et al., 1988; Kaup et al., 2005), viruses known to activate the cGAS–STING pathway (Sun et al., 2012; Gao et al., 2013a). RNA viruses, however, tend to induce mitochondrial cubic membranes (Schaffner et al., 1977) or form membranous cytoplasmic inclusions (Schaff et al., 1992). Strikingly, ER cubic membranes are found in lymph node tissues of almost all AIDS patients (Orenstein et al., 1983; Sidhu et al., 1983; Hammar et al., 1984), thus provoking much clinical and scientific interest. Nevertheless, the nature and pathogenesis of such structures remain obscure, although studies indicate a possible relation to the elevation of type I-IFNs (Luu et al., 1989), most likely via the activation of the cGAS–STING pathway. With the inspiration of STING phase-separator, more attention and efforts are needed to solve the long-lasting puzzles about the initiation and function of cubic membranes, especially those virus-related ones.

## Positive regulation of STING by PC7A–STING condensates

Besides the cGAMP-induced STING phase-separator, it is reported that a synthetic polyvalent STING agonist PC7A also induces liquid–liquid phase separation of STING (Li et al., 2021b). PC7A is a pH-sensitive polymer composed of a seven-membered ring with a tertiary amine. It activates STING through the polymer-induced formation of STING–PC7A condensates. PC7A binds to a non-competitive STING surface site that is different from the cGAMP-binding pocket, therefore prolonging STING activation and the downstream signaling. Different from the gel-like ER-located STING phase-separator, PC7A–STING condensate remains liquid-like state and translocates to the ERGIC–Golgi similar to the cGAMP-induced STING translocation, thus promoting STING accumulation, oligomerization, and activation. PC7A with higher degrees of polymerization induces PC7A–STING droplets with lower dynamic, which lead to decreased STING activation compared to the liquid-like PC7A–STING droplets. The phenomenon of suppressed STING activation in PC7A–STING condensates with lower dynamics is consistent with that in STING phase-separator. Still, more efforts are needed to elucidate the details of PC7A–STING condensates, including the mechanisms of its translocation, the final destination of the condensates, and membrane structures within the PC7A–STING condensate, particularly those on the TGN.

## Conclusions and perspectives

Our understanding of the regulation and activation of cGAS–STING signaling has advanced considerably over the past several years. The activity of cGAS is now revealed to be regulated by cytosol DNA, as well as metal ions including Zn^2+^ and Mn^2+^. As the cGAS–STING pathway is an important source of type I-IFNs, targeting cGAS by metal ions may offer tremendous therapeutic opportunities, such as immunotherapies and vaccine adjuvants (Figure 3). However, more works are still needed to fully understand the actions and side effects of Mn^2+^ in physiological and pathological conditions, as well as the process and mechanism of releasing Mn^2+^ from restrained cellular organelles during infection.

The activation and translocation of ER-membrane protein STING have remained one of the most enigmatic fields in innate immunity. Our review briefly introduces recent discoveries about structural advances in STING–TBK1 activation, COP-II/COP-I mediated STING ER–Golgi translocation, and sGAGs-mediated STING activation (Figure 3). The recently revealed agonist-binding pocket of STING at the transmembrane domain is quite adjacent to the binding sites of sGAGs. Since sGAGs are the second ligand of STING activation by facilitating STING oligomerization, it is quite possible that sGAGs are the natural ligand of STING transmembrane domain binding pocket. Structural studies of STING and sGAGs are in need to prove the hypothesis and elucidate the mechanisms of STING oligomerization in physiological conditions.

Cellular condensate formed by biologically regulated phase separation of biomolecules is now recognized as a fundamental mechanism for orchestrating physiological process, such as reaction kinetics or specificity by regulating molecular concentrations or sequestrating unwanted molecules. Recently discovered cGAS–DNA droplets, STING phase-separators, and PC7A–STING droplets indicate that the cGAS–STING pathway is also regulated in a manner of biomolecular condensation (Figure 4). Still, research on the regulation of innate immunity by biomolecular condensation has just begun and more attention is needed to elucidate the formation and regulation of cGAS droplets and STING phase-separators, as well as their function in physiological and pathological conditions. Also, due to the tight relationship between the STING phase-separator and cubic membranes, studies on the STING phase-separator indicate that previously discovered cubic membranes may also be a series of membranous biocondensate. Functional studies on the cubic membrane, especially the pathological ones, may need to focus on their physical properties to solve the long-lasting puzzles of their initiation and function.
